# History of the Origin, and Transactions of the Medical Societies of Buffalo

**Published:** 1861-09

**Authors:** Thomas F. Rochester


BUFFALO

### Medical and Surgical Journal

AND REPORTER.

VOL. I. SEPTEMBER, 1861. NO. 2.

### ORIGINAL COMMUNICATIONS.

ART. I.—*History of the Origin, and Transactions of the Medical Societies of Buffalo, by* THOMAS F. ROCHESTER, M. D.

The earliest record of Medical Association for professional improvement and advancement, to which access has been obtained, is entitled "The Constitution and By-Laws of the Medical Society of the Village of Buffalo, adopted July 16th, 1831." This is prefaced by the following preamble, which, with but slight alterations, has been adopted by each succeeding organization up to the present time:

"*Whereas,* medicine, as a science, is of great extent and difficult attainment, embracing a knowledge and implying a familiarity with the laws of Nature generally, and

*Whereas,* much may be done by a free and mutual interchange of medical opinions toward enlightening ourselves, and thereby benefitting the community in which we live, and

*Whereas,* The practice of our profession, at all times arduous and responsible, may, by an honorable and gentlemanly deportment, and a strict observance of professional courtesy, be rendered more agreeable to ourselves, more useful to our friends, and more dignified in the eyes of men, therefore the undersigned, practicing physicians and surgeons of the Village of Buffalo, do agree to associate for the promotion of the above laudable purposes, and to adopt the following Constitution and By-Laws by which they will be governed."

The Constitution consists of twelve articles, establishing the name and offices of the Association, and stating the number of officers and their several duties.

It is interesting to observe that the meetings of the Association, as established by Art. 9th, were to be "on the first Tuesday of every month"—the time corresponding with that of the meetings of the present organization.

Article X, is to the effect that all regular physicians, residing in the village, may become members, after a two-thirds vote in their favor, on signing the Constitution and By-Laws.

The By-Laws are thirty-six in number, and are all eminently useful and proper; many are both curious and minute, especially those pertaining to professional conduct and etiquette. By Section 3, absent members are fined, and by Section 9th, members who fail in their assigned duties are also fined heavily, and the records of the Secretary are to the effect that these fines were levied and paid. Section 13th is a full and, for the times, a respectable fee bill. Section 14th requires accounts to be presented and settled, if possible, once a year.

Section 15 reads, "It shall be incumbent upon every member of this Association to be courteous and gentlemanly, free from vulgarity of manners, habitual swearing, drunkenness, gambling, or any species of debauchery. It shall also be incumbent upon the members of this Association, when speaking of each other, to abstain from slander or detraction, or from stating any facts prejudicial to the character of a member, without first having presented the matter to the Association for their consideration."

Section 18 forbids self-laudation, or assumption of greater or special skill than any of the other members of the Association.

Section 25 says, "It shall be deemed unprofessional and savoring of quackery to introduce or encourage conversations with persons uninstructed in medicine, upon subjects relating to diseases, remedies, operations, and the like, or to expatiate upon the number of our patients, the frequency of calls, or to boast of great cures," &c.

Section 29 declares, "deductions from bills made out agreeably to the fee bill, as unfair and unprofessional, where the parties have the ability to pay the full amount."

Section 32 provides for the reproval, suspension or expulsion of members found guilty of dishonorable or ungentlemanly conduct, professional or otherwise; and Section 33 excludes expelled members from professional intercourse with the members of the Association.

Section 36 is a pledge of honorable agreement to the Constitution and By-Laws, and is signed by CYRENIUS CHAPIN, JUDAH BLISS, JOHN E. MARSHALL, J. TROWBRIDGE, MOSES BRISTOL, BRYANT BURWELL, HENRY R. STAGG, A. S. SPRAGUE, JAMES M. SMITH, LUCIAN W. CARYL, ORSON S. ST, JOHN.

On the 19th of July, 1831, the Association met for the purpose of finally adopting the Constitution and By-Laws and of choosing officers. The election resulted in the choice of

| | |
|---|---|
| Dr. CYRENIUS CHAPIN, - - | President. |
| " JUDAH BLISS, - - - - | Vice President. |
| " BRYANT BURWELL, - - | Recording Sec'y. |
| " JOSIAH TROWBRIDGE, - - | Corresponding Sec'y. |
| " MOSES BRISTOL, - - - | Treasurer. |

Six members were also appointed, by ballot, to read dissertations on specified subjects at successive meetings of the Association.

At the next meeting, August 2d, 1831, Dr. Caryl read his dissertation "On the Circulation," and Dr. Trowbridge "referred generally to the case of the lamented Mrs. Gen'l Porter, as one peculiarly and strikingly illustrating the impropriety of taking strong and drastic medicines in acute diseases, without the advice of skillful physicians."

During 1831 five regular meetings were held; two dissertations were read, and three members paid each two dollars forfeit, their papers not being prepared at the designated time.

On the second of January, 1832, a new election resulted in the choice of the following officers:

| | |
|---|---|
| JOHN E. MARSHALL, - - - | President. |
| BRYANT BURWELL, - - - | Vice President. |
| JOSIAH TROWBRIDGE, - - - | Corresponding Secreary. |
| LUCIAN W. CARYL, - - - | Recording Secretary. |
| ALDEN S. SPRAGUE, - - - | Treasurer. |

It was "Voted that Dr. Sprague send for the Boston Medical Journal, in the name and for the use of the Association."

It was also "Voted that the next regular meeting be held in the Hall of the Buffalo Lyceum."

*February 7th,* 1832, Dr. Bliss read his dissertation "on a case of Inverted Uterus."

*March* 6, 1832, the Association met and Dr. Chapin delivered his dissertation "On the Theory and Practice of Medicine, on the origin, nature and reatment of various diseases, especially of the Eastern Cholera," &c.

*April 3d*, *May 1st*, and *June 5th*, 1832, the Secretary, Dr. Caryl, reports no quorum, and registers fines against delinquent members.

Thus, after an existence of less than one year died, of Inanition, "The Medical Association of the Village of Buffalo."

The next record pertaining to Medical Association, is dated January 22d, 1836—when "At a meeting of the Physicians and Surgeons of the City of Buffalo, held at the office of Drs. Marshall and Harris, a resolution was offered by Dr. Marshall and passed,

"That it is deemed proper and expedient to establish fixed prices for medical services."

"Drs. Burwell, Marshall, Barnes, Hawley and Winne, were appointed a committee to draw up a list of charges and report the same."

A resolution was also offered by Dr. Winne,

"That a committee of five be elected by the physicians present to draw up a Constitution and Code of By-laws for a proposed Medical Association of the Physicians of the City of Buffalo.

"Drs. Miller, Salisbury, Sprague, McVicar and White, were elected such committee."

On the 27th of January, 1836, the adjourned meeting met at the same place, and Dr. Pratt was appointed Chairman, and Dr. Winne Secretary. The committee appointed to prepare a list of charges for medical services, reported a full Medical and Surgical Fee Bill, prefaced by a long preamble, setting forth the necessity of an increase on the rates adopted in 1831 based on the unexampled growth and prosperity of the now, City of Buffalo, and the consequent proportionably enlarged expenses of living. Fee bill adopted, and a pledge taken to adhere to it by Drs. Bryant Burwell, Henry R. Stagg, J. V. Hawley, John E. Marshall, C. H. Reynolds, Brock McVickar, Charles Winne, James P. White, Abraham Miller, Judah Bliss, Josiah Barnes, A. J. Sprague, F. L. Harris.

The committee appointed to draw up "a Constitution and Code of By-laws for a proposed Medical Association of the Physicians of the City of Buffalo," failed to report, and no organization was effected, because, as the writer is informed, by the mover of the resolution, "everybody was carried away by the spirit of speculation, engendered by the sudden prosperity of the times, and the place."

From this period, nearly ten years elapsed before the Buffalo Medical Association came into being. The following account of its "origin and formation," is a transcript from the first volume of its transactions.

"On the evening of *July 2d*, 1845, a meeting was held at the office of Dr. Josiah Trowbridge, pursuant to the following notice in the Buffalo *Medical Journal*, July 1st, 1845 :

"To the Physicians of Buffalo :—Physicians of this city, (members of the Erie County Medical Faculty,) who are disposed to unite in forming a City Medical Society, are requested to meet at the office of Dr. Josiah Trowbridge, on Wednesday evening, 2d inst., at 7 o'clock." The following gentlemen were present at this meeting—Drs. J. Trowbridge, Moses Bristol, A. S. Sprague, Geo. N. Burwell, John S. Trowbridge, Charles Winne, Josiah Barnes, F. L. Harris, H. N. Loomis, H. M. Congar, F. H. Hamilton and Austin Flint. The meeting was organized by calling Dr. J. Trowbridge to the Chair, Dr. Flint was appointed Secretary. The Chair having stated the object of the meeting, it was moved by Dr. Winne, that a society of the Physicians of the city be formed, which motion was seconded by Dr. Congar. After some discussion, the motion was put and carried. It was then moved by Dr. H. N. Loomis, that a committee of three be chosen to draft a Constitution and By-laws, and report at an adjourned meeting, to be held, in two weeks. This motion was seconded by Dr. Bristol, and carried. Drs. Loomis, Winne and Flint were chosen such committee. The meeting then adjourned to meet July 16th, which convened agreeably to the adjournment, at the office of Drs. Sprague and Loomis. Dr. Sprague presided, in the absence of Dr. Trowbridge. The minutes of the previous meeting were read, when it was moved by Dr. Winne, that they be so amended, that it shall appear that the motion made by him, as to forming a society, was for the purpose of expediting business, and that he was opposed to the formation of a society. Seconded by Dr. Flint and carried. In the absence of Dr. Loomis from the city, (Chairman of the Committee to draft a Constitution and By-laws,) and by request of Dr. L., Dr. Flint reported a Constitution and By-laws. Dr. Winne then made a verbal minority report, stating that he differed from the views which had originated the report by the majority of the committee."

The constitution and by-laws were, on motion of Dr. Pratt, taken up *seriatim*, and after two or three alterations, were adopted. Officers for the ensuing year were then elected as follows :

Josiah Trowbridge, M. D., - - President.
Alden S. Sprague, M. D., - - - Vice President.
Austin Flint, M. D., - - - Recording Secretary.

Those present then affixed their names to the constitution and by-laws, and the meeting adjourned to hold *the first* regular meeting of the Association at the office of Dr. F. H. Hamilton, on the 5th of August, 1845, at 8 P. M.

The constitution and by-laws were based upon those of the village society of 1831, and were signed by

J. Trowbridge, M. Bristol, James P. White, A. S. Sprague, H. H. Bissell, John J. Trowbridge, S. F. Mixer, G. N. Burwell, J. B. Samo, S. G. Bailey, Austin Flint, G. F. Pratt, F. L. Harris, H. M. Congar Wm. Treat, Silas Hubbard, Chas. H. Wilcox and Josiah Barnes—whole number 18.

We now come to the "Proceedings." Of these, but a mere abstract of the more important can be given for want of both time and space. Sufficient reference however will be made to reports of cases, and to discussions on professional subjects, to show the budding of the fruit, that has under the care of several of the members, been brought to deserved repute, and to present a brief chronological history of disease, of remedial agents and of opinions and events that bear upon them, and that have either a local or general interest or significance.

AUGUST 5TH, 1845—8 P. M.

The association met, according to adjournment, and Dr. Trowbridge, the President, on taking the Chair, read an able address, the greater portion of which was published in the Buffalo *Medical Journal*, which had been *first* issued in the preceding June. At the same meeting Dr. Flint presented for inspection a *heart*, with valvular lesions, and Dr. Hamilton moved the appointment of a committee "to collect statistical information concerning *fractured limbs, shortening, &c.*," and at the meeting next ensuing, Dr White presented a *placenta* with ossiffic deposit, and reported a case of ovarian dropsy, caused by a severe fall on the abdomen.

It is certainly very gratifying to find in the first records of the proceedings of the Association, the manifestation of those proclivities, which subsequently carried out, have stamped so honorably, in the medical world, the gentlemen mentioned, and while their lustre is reflected upon the Association, the latter may claim that but for the field afforded by it, the repute they have so deservedly gained, might never have been sought for.

At the October meeting, Dr. Trowbridge, stated the *now* well established fact, "that extensive organic disease of the stomach often existed without much functional derangement." A discussion then ensued upon the external use of Tr. of Iodine in various affections. From the tenor of the remarks, it is inferred that up to this period it had been but little employed.

*Dec.* 3*d*, 1845.—A committee, composed of Drs. White, Barnes and Flint, reported a fee bill, which was adopted and ordered printed for the use of the members.

*January* 6, 1845.—Dr. Flint reported a case of "true typhoid fever."

*February* 6, 1846.—Several cases of typhoid fever were reported, and a discussion on treatment ensued. Dr. J. Trowbridge remarked that typhoid fever had not prevailed here until lately, but that typhus fever had occasionally appeared for many years, and stated that in his judgment, the former affection should not be treated heroically. Dr. White was not in favor of much medication, but preferred to sustain and nourish the patient.

At the same meeting Dr. Hamilton, speaking of enlarged tonsils, remarked that he had removed them from 150 to 200 persons, and had only lost one patient, who really died from scarlatina, one week after the operation. A similar instance was reported by Dr. Sprague ; bleeding from the tonsil being coincident with the eruption, which appeared five hours after the excision of the gland. Death ensued in 24 hours.

Toward the close of the meeting, "under the provision of the constitution, for the impeachment of members," Dr. A—— made a statement of occurrences, in which he attributed to Dr. B——, unfair and unprofessional conduct toward himself. Dr. B., made a counter statement, and disclaimed any intention of committing a breach of medical etiquette. Remarks were made by several members, pro and con, when Dr. C—— moved the following resolution :

"*Resolved,* That in view of the mutual statement made by Drs. A., and B., the association advise the parties to hold the matter no longer the occasion of unpleasant feelings."—Carried unanimously.

This incident is mentioned as suggestive of one of the advantages of medical societies—the prevention or the removal of professional differences.

*March* 3*d*, 1846.—Dr. J. S. Trowbridge reported Typhoid Fever at the Alms House, and attributed its origin there to emanations from the cellar which contained stagnant matter and much filth.

Dr. B. Burwell reported a case of very severe Erysipelas in an elderly lady of feeble habits, treated by copious and repeated blood-letting, and by the administration of calomel, opium and antimony; he ascribed her recovery to the active measures employed.

Dr. Flint was adverse to depletion in erysipelas, from considerations of general pathology, and reported two cases treated exclusively with quinine the results sustaining this method of treatment.

The Secretary applied for permission to publish the proceedings of the Association in the Buffalo Medical Journal. Granted.

Dr. B. Burwell introduced some resolutions relating to the National Medical Convention to be held in New York, May 1st, 1846, to the effect that such a Convention is useful and necessary, and that County Societies and Medical Associations should be represented therein.—Carried. A copy of the resolutions was directed to be sent to the President of every Medical College in the State. At a subsequent meeting Dr. B. Burwell and Dr. A. S. Sprague were elected delegates to the first National Medical Convention.

*Nov.* 3, 1846, Dr. Sprague reported a case of puerperal eclampsia, pos partum, relieved by the administration of quinine.

April 6, 1847.—Remarks were made upon the "inhalation of sulphuric ether and its anæsthetic properties;" by whom, not stated.

*April* 27, 1847.—It was moved by Dr. White that the sum of $25 be raised by subscription toward defraying the expenses of the delegate to the National Medical Association.—Carried. Dr. J. Trowbridge was elected delegate, but declined serving. A committee was then appointed to select a delegate, but reported subsequently that they had failed to do so.

*Aug.* 3d, 1847—Election—Dr. B. BURWELL, President; Dr. C. H. AUSTIN, Vice President; Dr. W. TREAT, Secretary.

*Sept.* 7, 1847.—The Society adopted the code of ethics recommended by the National Medical Association.

The By-Laws, according to previous notice, were amended, providing for the appointment of a Primary Board.

*January* 4, 1848.—Dr. Sprague reported the successful amputation of a thigh, the patient being etherized and unconscious throughout the operation.

*Feb'y* 1st, 1848.—Dr. Hamilton related the effects of chloroform obtained from Boston: he had inhaled about 1 oz.

Dr. Lockwood had seen a patient very ill and faint, requiring stimulants for three days, after inhaling a very small quantity preparatory to the extraction of a tooth.

The question of the employment of ether in obstetric practice was then discussed, and elicited an opinion from the meeting unfavorable to its use. Dr. Loomis thought well of it in surgical operations, if employed with proper care.

*April* 4, 1848.—Dr. Hamilton offered the following preamble and resolutions:

As a memorial to ourselves and our successors, of the first President of this Association, and of one venerable and justly distinguished in his profession, whose excellent counsels and unvarying professional courtesy have long commanded our profoundest respect and admiration,

Resolved, That measures be immediately taken to procure for this Association a faithful portrait of Dr. Josiah Trowbridge.

Resolved, That a Committee of five be appointed to wait upon Dr. Trowbridge and ascertain whether he can comply with our wishes, and if so, at what time it will be convenient for him to do it.

Resolved, That the Committee be further authorized to engage for this purpose a competent artist.

Adopted, and the Chair appointed Drs. Hamilton, Treat, Sprague, Congar and Bristol, the Committee for purposes named.

(The portrait, as it subsequently appears, was painted by Wilgus, to whom the sum of $68 was paid. The portrait was deposited in the Common Council Room, November 7, 1848, where it remained until proper rooms were secured by the Society.)

The resolutions offered by Dr. Hamilton having been passed, Dr. G. N. Burwell reported seven cases of obstetrics in which he had used chloroform with entire safety, and to the great comfort of the patients.

On motion of Dr. Flint, the Society instructed its delegate (Dr. W. Cary) to invite the National Medical Association to hold its next Convention in this City.

*May* 2d, 1848.—On motion of Dr. White the By-Laws were amended making the first Tuesday in April the time for the annual election of officers. This was done that their names might be printed in the City Directory which was issued early in June every year.

*June* 6, 1848, (pp. 89, 90,)—Dr. Congar made a long delayed report on *vaccine*, or rather on a vaccine depot. A. I. Mathews was elected keeper of the same.

Dr. Hamilton reported a case of vesical flatulence in a male adult, offensive gas being discharged from the meatus urinarius, and Dr. Hamliton also reported that a man suffering with obstinate constipation, took to his knowledge, 1 lb. of crude quicksilver. It had been retained for two days, and was still in statu quo. Thus we have on record, what will one day be regarded as a therapeutic myth.

Dr. White, as Chairman of Committee, reported in favor of renting a room for the Society, at a cost not exceeding $50 per annum. The Committee was discharged, and the consideration of the subject postponed.

Election being in order, the following officers were chosen to serve from date to the first Tuesday in April ensuing:

| | |
|---|---|
| Dr. F. H. Hamilton, - - - - | President. |
| " S. F. Mixer, - - - - | Vice President. |
| " William Treat, - - - - | Secretary. |
| " Flint, Cary and Congar, - - | Primary Board. |

*Oct.* 3*d*, 1848.—Patent medicines were discussed, and H. O. Hayes, druggist, was highly commended, having abandoned the sale of these nostrums.

*Dec.* 5, 1848.—A Committee was appointed to prepare a popular article on *Cholera*, including sanitary and preventive measures, to be published in the daily journals. The report was presented and adopted December 13th.

*April* 3*d*, 1849—Election—S. F. Mixer, President; G. N. Burwell, Vice President; James M. Newman, Secretary.

Dr. White reported a case of deficiency of abdominal wall in a child born at seven months' gestation. "The funis, at the surface of the body, was expanded to the size of a closed hand, was translucent, and the peristaltic movement of the bowels was visible; a pad and bandage constituted the dressing, and three weeks after birth the orifice had contracted to the size of a sixpence, and the child was doing well."

*May* 1*st*, 1849.—Dr. Samo, from Committee appointed for that purpose, made a long and able report, "recommending measures for a uniform and equitable compensation for medical officers appointed in the city and county," suggesting the following as the minimum salary for the several duties and places:

| | |
|---|---|
| Alms House, - - - - | $1000 per annum. |
| Jail, - - - - - | 100 " |
| Work House, - - - - | 200 " |
| Coroner's cases, - - - - | 3 to $5 each. |

Attendance on Cholera Hospital, each $5 per diem.

The Report was referred to a joint Committee of the City and County Societies.

*June* 5, 1849.—Dr. Cary reported a case of Asiatic Cholera, the first in Buffalo during the epidemic.

*July* 10, 1849, Tuesday evening, Special Meeting.—"Dr. J. Trowbridge stated that the meeting had been called for the purpose of establishing a uniformity in reporting cases of the prevailing *Epidemic Cholera* to the

Board of Health, and to ascertain by conference with the Board of Health, what in their view were cases of cholera, and what they expected physicians to report as cases of such disease."

Dr. C. C. Haddock and Mr. A. McArthur, members of the Board of Health of the City of Buffalo were present. Dr. Haddock stated "that the Board had assumed the position that in all cases where the biliary secretions were checked, with colliquative discharges, and with checked secretions of the secretory organs generally, were cholera."

On motion of Dr. White, it was unanimously resolved, that a committee of three be appointed by the Association to draft a report expressive of the sense of the Association, relative to the objects of this meeting, and who shall report at an adjourned meeting on Thursday evening next. Drs. Flint, Treat and J. S. Trowbridge, were appointed such Committee.

The following note is appended to the record of the proceedings by the Secretary:

"On Wednesday morning—the next morning after the above meeting—Dr. C. C. Haddock, who was then present from the Board of Health, in apparent good health, was attacked with the *diarrhœa*, which in his anxiety for the good of others, and from the increased labors in the discharge of his duties as Health Officer, he neglected to attend to, until late in the evening of the same day, at which time he was attacked with the *cholera*, and died the next afternoon, some hours previous to the time of the assembling of the meeting adjourned as above."

The adjourned meeting was held in the Common Council Room, July 12, 1849; his Honor, Mayor Barton, and Mr. A. McArthur, from the Board of Health, being present.

Dr. Flint, Chairman of the Committee on Cholera, read a very able and elaborate report, which was printed in the 5th vol., 3d number Buffalo Medical Journal, August, 1849, and a copy of the same was ordered presented to the Board of Health.

The death of Dr. Haddock, a member of the Board of Health, was then announced by Dr. J. S. Trowbridge, and suitable resolutions were passed on the sad event.

The next item of general and local interest is found in the records of Jan. 8th, 1850. "Dr. Harvey, (dental surgeon,) was asked his opinion, of the possibility of a dentist identifying a jaw bone, as is said to have been done by Dr. Keef, in the late case of Dr. Parkman, of Boston. He thought it impossible, if the flesh was denuded from the jaw, and the plate not procured in a perfect state."

*April 2d,* 1850—Election—President, Dr. G. N. BURWELL ; Vice President, Dr. C. W. HARVEY ; Secretary, Dr. WM. Ring.

*Sept. 3d,* 1850.—Dr. Hamilton read a biographical sketch of the late Dr. Marshal.

*Oct. 1st,* 1850.—" Drs. Burwell, Pratt and Treat were appointed a committee to prepare a biography of the late Dr. Chapin of Buffalo."

*April 1st,* 1851—Election.—President, Dr. C. W. HARVEY ; Vice President, Dr. SILAS HUBBARD ; Secretary, JAMES S. HAWLEY.

Primary Board—Drs. Wallace, Wyckoff and Treat.

*May* 6, 1851.—Drs. Ring, Sprague and Hamilton, were appointed a committee to obtain signatures to a petition to the State Legislature in favor of legalizing dissections.

A discussion arising as to the propriety of blood-letting in scarlet fever, and other acute diseases, Dr. Trowbridge was of the opinion that the profession resort less to blood-letting now than formerly, owing to the improved state of our knowledge of disease and its treatment. The writer has thought this opinion as especially worthy of mention, as coming from one, whose age and experience entitle it to much weight, and particularly as it opposes the too generally received idea, that it is due to a change in the character of disease itself.

On motion of Dr. Strong, Drs. Burwell, Strong and Congar were appointed a committee to memorialize the Common Council in relation to the more perfect registration of deaths and their causes.

The Association at this meeting, resolved to sit every fortnight instead of monthly, as heretofore.

*June* 17, 1851.—The Secretary was authorized to assess a tax of fifty cents upon each member for the current expenses of the year.

*Dec. 3d,* 1851.—Dr. Bailey stated that he had recently reduced a dislocated femur by Dr. Reid's method, and found it easy and successful.

Dr. Palmer said dissections of the muscles of the thigh showed the method to be philosophical.

*April* 6, 1852.—" A report of cases illustrating the localization of valvular lesions of the heart," by Dr. Flint, was presented and accepted by the Association.

Election—President, Dr. C. W. HARVEY, (re-elected ;) Vice President, Dr. SILAS HUBBARD, (re-elected ;) Secretary, Dr. J. EASTMAN.

Primary Board—Drs. Wallis. Strong and Garvin.

*July* 6, 1852.—" It being the evening for the funeral obsequies of Hon.

Henry Clay, on motion of Dr. Eastman, the Association adjourned for one week."

*Aug. 3d,* 1852.—Another record of cholera—sixty-two cases and thirty deaths, within the preceding week. A ditch on Ellicott street, opened by the Water Works Company, for laying pipe, caused much of this sickness and mortality.

Drs. Hamilton, Wilcox and Strong, were appointed to report on the relation of the upturning of the soil, to the causation of cholera.

*Sept.* 7, 1852.—Provision was made for the election of honorary members.

*Nov.* 9, 1852.—Dr. White presented, for inspection, a cast of the male organs of generation, with the uterus and ovaries attached, the vagina opening into the prostatic portion of the urethra, patient menstruated regularly, but was unable to procreate, the testes being within the abdominal walls.

Dr. S. E. S. H. Nott, then contributed to the amusement of the members of the Association, at the expense of their credulity, by the narration of two extraordinary (!) cases, ending in recovery.

*The first,* that of a man, who was submerged in a mud hole, two feet deep, under a capsized load of wood, for at least half an hour. When the wood was removed, the body was not visible, but exploration detected it. When it had been pryed out, it was held up by the heels, and evacuated by the mouth, a pailful of mud and water. The *corpus* was then allowed to assume the dorsal decubitus, and a bottle of whiskey was applied to the labial commissure. It proved a "method" of resuscitation "more ready' than that of the illustrious Marshall Hall, while it illustrated his theory of *reflex action* perfectly, for no sooner was the labial contact established, than it was followed by the expansion of the orbicularis and by powerful contractions of the muscles of deglutition.

*The second,* that of a prostitute, who, with suicidal intent, swallowed half an ounce of arsenious acid. Indignant nature, after three hours and a half of tolerance, repudiated the assault by active emesis. Twenty-four hours afterwards *medical science!* came to dame nature's aid with morphine, barley water, and sulphate of magnesia, the nymph was rescued, and was soon in condition to resume her avocation.

*Dec.* 7, 1852.—There was a discussion on the use of opium and its preparations in the treatment of diarrhœa, dysentery, cholera, cholera morbus, colic and peritonitis. Many instances of its efficacy and tolerance were brought forward, especially by Drs. White, Flint and Strong; the former mentioned with appropriate comments the case of puerperal peritonitis, successfully treated by Dr. Alonzo Clark of New York, in which 280 grains of opium were administered in twenty-four hours.

*March* 22, 1853.—By resolution, the portrait of Dr. Trowbridge was removed from the Common Council Chamber to the rooms of the "Young Men's Association," to be temporarily under the charge of the "Committee on Local History and Fine Arts."

*April* 5th, 1853—Election—President, Dr. SAMUEL CARY, (declines;) President, Dr. C. H. WILCOX; Vice President, Dr. J. M. NEWMAN; Secretary, Dr. J. ROOT; Primary Board, Drs. EASTMAN, HAWLEY and RING.

*June* 21, 1853.—Extracts from the records: "Dr. Flint reported the number of fatal cases occurring in his practice of all diseases, during the summer of 1852. Dr. F. also stated the reasons of this report. He had reason to know from the best authority that certain medical men of this city, and also members of this Association, had industriously reported among the community, that he had been very unsuccessful in his practice, and that his mode of treatment was unusual. These statements he, Dr. F. wished to show were maliciously false, for he held that a man who reported a slander which he did not *know* to be true, was equally base with him who slandered with a design to injure. These false statements had been made not only in Buffalo, but also in a neighboring State, where he was delivering a course of lectures during the past winter. These records of the fatal cases in his practice were made not to defend his mode of treatment or with a view to publication." The report was, on motion, ordered to be entered on the records of the Association, [pp. 197, 198.]

The above extract is given in full, as illustrating the necessity of a Medical Association, as a tribunal, before which all honorable physicians may present their professional grievances.

*July* 5, 1853.—"Dr. Marshall Hall, of London, in reply to a letter directed to him at the Falls, by Dr. Flint and the President of the Association, inviting him to meet the Association at some convenient time, replied that he would do so on Thursday evening next, and would lecture on epilepsy or the reflex nervous system. Drs. Flint, Strong, Hamilton, Congar and White, were appointed a Committee of Arrangements."

*July* 7, 1853.—Association met at the residence of Dr. Flint. Dr. Marshall Hall proceeded to demonstrate the reflex nervous system on the frog, after which the evening was spent in social intercourse at the board of Dr. Flint.

*Nov.* 15, 1853.—Twenty-three members were present at a regular meeting. This is mentioned as illustrating the interest taken in the proceedings by the physicians of the City.

*Jan'y* 3*d*, 1854.—The present fee bill of the Buffalo City Association, now in force, [1861] was adopted.

*March* 7, 1854.—Dr. Hunt brought before the Society the necessity of taking observations of the *temperature* and *humidity* of the air at the same time, and their connection with diseases. Proceedings were at once instituted to obtain the proper instruments; and here started the valuable "hygrometric observations" that subsequently enrolled the name of Dr. Hunt among the reliable scientific enquirers of our country.

*April* 4, 1854—Election—President, J. M. NEWMAN; Vice President P. H. STRONG; Secretary, S. B. HUNT.

Dr. G. N. Burwell reported a case of hydrophobia from the bite of a cat, terminating fatally. The case of Dr. S. Hawley, a member of the Association, who was subsequently bitten by the same rabid animal, was also detailed. The Doctor's hand was badly wounded, although protected by a thickly-lined fur glove. The injured parts were thoroughly excised, and no symptoms of the disease were ever developed, (nor now, 1861, T. F. R.)

*June* 27, 1854.—Dr. Rochester reported a case of cholera, as having occurred June 12, 1854; the first, as far as known, this year. Dr. Hunt spoke of the epidemic *varicella* at and around Hornellsville. There had been three hundred cases and ten deaths. It was pronounced by many *variola.* Dr. Hamilton had seen two of the cases. They were unquestionably varicella.

*August* 1*st*, 1854.—Reports of many cases of cholera in Buffalo and at Niagara Falls and Suspension Bridge. The great fatality and rapidity of the disease at the latter place, was ascribed to the upturning of a large amount of soil. Several instances were reported in which the affection was fully and instantaneously developed, without any premonitory diarrhœa. Drs. Hunt and Fred. Gardiner had each spent a week, and Drs. Hamilton and Rochester several hours at "the Bridge," and all concurred in this latter statement. Dr. Hunt made some extended remarks illustrating the opinion that the changes observed in the prevalence and intensity of cholera, corresponded with the hygrometric variations in the atmosphere. "The higher the dew point the more severe the disease."

*Oct.* 3*d*, 1854.—Dr. Newman read a memoir upon "Congestion of the

Brain in Cholera," which, by request of the Association, was sent to the Buffalo Medical Journal for publication.

*Dec.* 5, 1854.—Dr. Baker said that his partner, Dr. T. T. Lockwood, "recently brought home an ovum [human] the size of a turkey's egg, on account of its beauty, the fœtus being enclosed in the amnion. It was brought in a cloth and laid on a table in a cold room; on looking at it he saw the fœtus first draw up one leg, and afterwards move both legs and arms distinctly, with a quick, sharp motion. Drs. Lockwood, King and Newell also saw it in motion an hour and a half after its expulsion. Dr. White saw it three hours after expulsion, the motion still very active. [The writer suggests this as a good case for medical jurisprudence, on the subject of criminal abortion.]

*Tuesday, February* 6, 1854.—A supper and social entertainment was given to the members of the Association by Dr. Bailey.

*March* 6, 1855.—A By-Law presented by Dr. White, in the January meeting, was passed unanimously, as follows: "Any member neglecting to attend the regular meetings of this Association, for twelve meetings consecutively, and remaining a resident of this City during that time, shall forfeit his membership." It was subsequently amended by the addition of "provided he does not pay his yearly dues." The Secretary was instructed to serve a copy of the new By-Law on those members not present at this meeting.

*April 3d,* 1855.—Dr. Hawley reported a fatal case of hydrophobia, also visited by several members of the Association. Tracheotomy, as advised by Marshall Hall, was made, but with no relief. Dr. White reported a case of cyanosis, with drawings of the cardiac malformation. It was ordered published in the Buffalo Medical Journal.

*Election.*—President, Dr. STRONG; Vice President, Dr. EASTMAN; Secretary, Dr. HUNT; Primary Board, Drs. HAWLEY, ROOT and BAKER.

*June* 5, 1855.—Dr. G. N. Burwell reported several cases of pneumonia, successfully treated by repeated venesection. Dr. Wyckoff, one by the same method. Dr. Newman, one equally severe and equally successful, "where the necessity or the propriety of venesection did not suggest itself."

*July 3d,* 1855.—Dr. G. N. Burwell read an able and elaborate paper on pneumonia, with statistics made up from the mortuary records of New York and Philadelphia—he also reported two additional cases in his own practice, since the last meeting. The propriety of the treatment advised and adopted, was warmly discussed pro and con, by several of those present, and as the subject was evidently one of great interest, it was moved by Dr. White, "that the subject of pneumonia, its pathology, prognosis and treatment, together with the diagnostic value of the buffy coat, and also the papers read upon this subject at this and the preceding meeting, be referred to a committee of three to report at the next meeting."—Carried Drs. Flint, Burwell and Gay, Committee.

Dr. Newman offered the following :

*Resolved*, That a Committee of three be appointed for the purpose of taking into consideration of renting and fitting up a suitable room for the meetings of this Association, and to ascertain if a sufficient sum can be raised among the members, for the proper furnishing of the same, and also to ascertain the amount of yearly subscription that can be obtained for its subsequent support.—Unanimously adopted.

Drs. Newman, Wyckoff and Wilcox, Committee.

*Tuesday Evening, Sept. 4th*, 1855.—Association met at the new room, which, since the last meeting, had been neatly and conveniently fitted up for its use, under the direction of the Committee on Rooms and Fixtures.

Dr. Flint, from the Committee on Pneumonia, stated that they had agreed to present separate reports, and that his own being the longest, would be read last. They were then severally presented by Drs. Gay, Burwell and Flint. The papers were able and elaborate, and elicited from the members of the Association much commendation. These and the discussions and correspondence to which they led, were published in the Buffalo *Medical Journal*. They were extensively copied and commented upon by other medical periodicals, and were universally regarded by those journals and by the profession abroad, as evidences of the useful and elevated position of the Buffalo Medical Association.

[TO BE CONTINUED.]

